# Assessment of the Biological Activity and Phenolic Composition of Ethanol Extracts of Pomegranate (*Punica granatum* L.) Peels

**DOI:** 10.3390/molecules25245916

**Published:** 2020-12-14

**Authors:** Željka Peršurić, Lara Saftić Martinović, Mladenka Malenica, Ivana Gobin, Sandra Pedisić, Verica Dragović-Uzelac, Sandra Kraljević Pavelić

**Affiliations:** 1 Department of Biotechnology, University of Rijeka, Radmile Matejčić 2, HR-51000 Rijeka, Croatia; zpersuric@biotech.uniri.hr (Ž.P.); lara.saftic@biotech.uniri.hr (L.S.M.); 2 Faculty of Medicine, University of Rijeka, Braće Branchetta 20, HR-51000 Rijeka, Croatia; mladenkams@medri.uniri.hr (M.M.); ivana.gobin@medri.uniri.hr (I.G.); 3 Faculty of Food Technology and Biotechnology, University of Zagreb, Pierottijeva 6, HR-10000 Zagreb, Croatia; spedisic@pbf.hr (S.P.); vdragov@pbf.hr (V.D.-U.); 4 Faculty of Health Studies, University of Rijeka, Viktora Cara Emina 5, HR-51000 Rijeka, Croatia

**Keywords:** phytochemicals, pomegranate peel, antioxidant activity, antibacterial activity, antiproliferative activity

## Abstract

Pomegranate (*Punica granatum* L.) is a rich source of constituents with confirmed strong biological activities. However, pomegranate peel, which encompasses approximately 30–40% of its weight, is treated as a biological waste. The aim of this paper was to evaluate the potential of pomegranate peel extracts and to propose its functional properties that can be used for development of functional products. Eight ethanol extracts of pomegranate peels (PPEs) were characterized by use of direct infusion quadrupole-time of flight (Q-TOF), and afterwards tested on their antioxidant, antibacterial and antiproliferative activities. Mass spectrometry analysis revealed that the most prevalent compounds in pomegranate peels were punicalagin, granatin and their derivatives. Analysed extracts had high total phenolic contents that ranged from 5766.44 to 10599.43 mg GAE/100 g, and strong antioxidant activity (7551.31–7875.42 and 100.25–176.60 μmol TE/100 g for DPPH and FRAP assays, respectively). The results of biological activity assays showed that all PPEs possessed antibacterial activity, and that *S. aureus* was the most sensitive specie with minimum inhibitory concentration and minimum bactericidal concentrations ranging from 0.8 to 6.4 mg/mL. Additionally, the analysis of antiproliferative activity revealed high potency of PPEs, as the IC50 values ranged from 0.132 mg/mL to 0.396 mg/mL. Multivariate analysis pointed out the most discriminative metabolites for antioxidant or antiproliferative activity. Overall, the pomegranate peel confirmed to be a highly valuable source of bioactive compounds that could be used to improve the food functional characteristics.

## 1. Introduction

Pomegranate, lat. *Punica granatum* L., is one of the oldest fruits, belonging to the group of the first cultivated fruits, along with grapes, olives, figs and dates. In addition, it belongs to the group of the fruits that possesses the strongest beneficial pharmaceutical effects, mainly due to high concentration of diverse bioactive constituents [1].

In the production of pomegranate juice, which is obtained by squeezing seeds, the pomegranate peel is a waste product. Usually, after industrial processing, pomegranate peel is used as an animal feed. Since pomegranate peel has good nutritional and antioxidant properties, studies show that feeding cattle with pomegranate peel significantly improves the nutrition of livestock and improves their health [2]. However, pomegranate peel could have an even wider application. Namely, previous studies showed that, in comparison with other pomegranate fruit parts, peel contains a high concentration of phenolic compounds, with hydrolysable tannins as main compounds [3,4]. For example, Alexandre et al. proposed high pressure extracts from pomegranate peel as a source of added-value biologically active compounds for application in food matrixes to increase antioxidant activity and to reduce the risk of pathogenic contamination [5]. The addition of pomegranate peels extracts was successfully tested in yoghurt samples to increase its antioxidant content [6], in meat product to improve its oxidative stability [7] and in fruits to protect it from mycotoxigenic fungi [8]. Therefore, the aim of this study was to determine functional potential of pomegranate peel ethanol extracts and to connect specific metabolites with the desired biological activity.

The number of studies on tumours and multidrug-resistant bacteria has increased in recent years, and scientists are increasingly focusing on natural compounds that have a potential biological impact. Finding such compounds in biomass, that is considered as a biological waste, would be of great importance, mainly because large quantities of starting material would be easily reachable and in the same time, management of biological waste would be improved.

In the process of evaluating the potential of pomegranate peels, we firstly determined the phytochemical profile by use of direct infusion quadrupole-time of flight (Q-TOF) mass spectrometry (MS), and secondly investigated its antioxidant, antiproliferative and antimicrobial activities. The antiproliferative potency of ethanol extracts of pomegranate peels (PPEs) (*Punica granatum* L.) were tested on the WI-38 (normal human fibroblasts), MCF-7 (metastatic adenocarcinoma of the breast), HeLa (cervical cancer), MIA PaC-2 (pancreatic tumour tissue), CFPAC-1 (pancreatic adenocarcinoma, derived from metastatic liver) and SW620 (colon cancer adenocarcinoma) in vitro. Additionally, antibacterial activity was tested on *Staphylococcus aureus* ATCC 25923, *Escherichia coli* (ATCC 25922), *Acinetobacter baumannii* (ATCC 19606 and ATCC BAA-1605), *Listeria monocitogenes* (ATCC 19115), *Pseudomonas aeruginosa* (ATCC 27853) and *Enterococcus fecalis* ATCC 29212. Afterwards, all data were statistically processed to obtain clustering patterns of the samples according to the biological activity and to determine the specific components in pomegranate peel extracts that positively influence desired biological activities.

## 2. Results and Discussion

### 2.1. Chemical Composition

For characterization of the P1–P8 PPEs, four different methods were applied. First, for all extracts, direct infusion Q-TOF MS scan analysis in positive and negative ion modes was applied in order to generate metabolite profile specific for each sample. Afterwards, for all samples direct infusion Q-TOF MS/MS analysis was performed in order to identify components of PPEs. Results of the analysis are shown in the Table 1, whereas it can be seen that majority of identified bioactive compounds are from the group of tannins (ellagitannins and ellagic acid derivatives). These results are in accordance with previously published data [9]. Namely, in all the extracts, punicalagins, ellagic acid pentose or hexose derivatives and punicalin were identified (Table 2). Interestingly, some of the compounds were present only in few pomegranate peel samples, such as casuarinin, digalloyl hexose, epigallocatechin, lergastannin C, peducalangin II, peducalangin III and granatin like compound. For some of the compounds full identification was not possible and their tentative identification was carried out by comparison of the obtained fragments with the common pattern of fragmentation for ellagitannin group of compounds. This group of compounds possess hexahydroxydiphenoyl (HHDP), galloyl and glucose chemical entities and can be characterized due to the specific *m*/*z* losses during fragmentation process [3,10,11,12,13,14,15]. For example, for compound *m*/*z* 938.1025, due to the specific fragments [785.0197]^-^ and [300.9799]^-^ that correspond to the ellagitannin family of compounds (granatin compounds), tentative identification was granatin like compound. The obtained data were afterwards used for chemometric analysis in order to reveal the specific compounds that are responsible for analysed biological activity.

### 2.2. Antioxidative Activity and Total Phenolic Content (TPC)

Free radical attack is thought to play an important role in the aging process and progression of diseases such as cardiovascular disease, tumour and neurodegenerative diseases [16]. Therefore, one group of the methods used for determination of the pomegranate peel functional potential encompassed antioxidant assays: DPPH and FRAP. Additionally, for all extracts total phenolic content (TPC) was determined to quantify these bioactive compounds and to find eventual correlation with antioxidative activity. Results of the TPC, determined with the Folin-Ciocalteu approach, and antioxidant activities are shown in the Table 3. High values for all the analyses can be observed. Values for the TPC are ranging from 5766.44 ± 114.86 to the highest of 10599.43 ± 17.57 mg GAE/100 g among samples. In contrast, antioxidant activity obtained by DPPH assay showed similar results among extracts with results ranging from 7525.42 ± 70.71 to 7831.42 ± 12.59 μmol TE/100 g. FRAP assay showed higher variability among samples. The lowest antioxidant activity was obtained for the sample P1 (100.25 ± 0.26 μmol TE/100 g), and the highest detected was 176.60 ± 3.47 μmol TE/100 g for the sample P4. Correlation between TPC and DPPH or FRAP data was observed (R^2^ 0.85 and 0.82, respectively), thus, pointing out importance of phenolic compounds for the antioxidant capacity of pomegranate peels.

### 2.3. Antiproliferative Activity

Antiproliferative effects of ethanol extract of pomegranate peels on WI-38 (normal human fibroblasts), HeLa (cervical carcinoma), SW620 (colorectal adenocarcinoma, metastatic), MCF-7 (breast epithelial adenocarcinoma, metastatic), MIA PaCa-2 (pancreatic carcinoma) and CFPAC-1 (pancreatic adenocarcinoma, derived from metastatic liver) were tested *in vitro*. Chosen tumour lines encompass each type of tissue and varying degrees of invasiveness and potential for metastasis. Previous studies that documented the effective impact of pomegranate on tumour cell lines gave an appropriate rationale for testing antiproliferative activity [17,18,19,20,21].

Antiproliferative effects were observed for all eight samples in vitro (Table 4). The IC_50_ values obtained varied between samples and cell lines in the range from 0.132 mg/mL to 0.396 mg/mL. The lowest concentration required to inhibit 50% of the pancreatic carcinoma cell line MIA PaCa-2 was observed by the activity of the extract from the sample P6 and was 0.132 mg/mL. Previously, it was shown that pomegranate extract from the whole fruit effectively inhibited growth and viability of human pancreatic cancer cells by inducing cell cycle arrest [22]. Still, the main compound from the extract that contributed to the observed anticancer activity was not identified, pointing to the need for further investigations aimed to elucidate the chemical composition of pomegranate. Similarly, Kim et al. showed that different pomegranate fractions have multiple breast cancer suppressive activities [17]. In this study, ethanol extract of pomegranate peels also showed inhibitory effect on the proliferation of MCF-7 breast cancer cells with the IC_50_ values in the range from 0.162 to 0.303 mg/mL. These results are in accordance to the results obtained by Nazeam et al., where IC_50_ for aqueous, 50 % methanolic and 100 % methanolic fractions of pomegranate husks were 0.249, 0.285 and 0.179 mg/mL, respectively [23]. The inhibitory concentrations of analysed PPEs on cervical carcinoma (HeLa) ranged from 0.141 to 0.212 mg/mL which may probably be correlated with the presence of ellagic acid according to the literature where the ellagic acid from pomegranate had a promising inhibitory effect on the growth of human cervical carcinoma cells [24]. Inhibition of 50% of cells for normal fibroblasts WI-38 was obtained at higher concentration for P6 extract (0.319 mg/mL), confirming the selectivity of this sample towards inhibition of tumour cells growth. Even though analysed PPEs showed antiproliferative activity on different tested tumour cell lines, additional tests are needed if the molecular mechanisms underlying the observed antiproliferative activity is to be elucidated.

### 2.4. Antimicrobial Activity

PPEs were additionally tested for their antibacterial properties and the results are presented in the Table 5. According to bacterial susceptibility to all tested PPEs, *S. aureus* was the most sensitive species with MIC ranged from 0.8 to 6.4 mg/mL, and MBC ranged from 0.8 to 6.4 mg/mL. These results are in accordance to results obtained by Voravuthikunchai and Kitpipit where MBCs of ethanolic extract of pomegranate peels for *S. aureus* ranged from 0.4 mg/mL to 1.6 mg/mL [25]. In contrast, another Gram-positive bacterium, *L. monocytogenes*, revealed the minimum sensitivity to tested PPEs with MIC and MBC of 12.8 mg/mL for all samples. As for the antibiotic sensitive and resistant strains of *A. baumannii*, sensitive strain ATCC 19606 showed greater sensitivity to the all tested extracts with MIC ranged from 3.2 to 6.4 mg/mL, and MBC ranged from 6.4 to 12.8 mg/mL. Regarding multiresistant strain ATCC BAA-1605, all tested extracts inhibited the growth, while only samples P1, P4 and P6 were bactericidal. Gram negative bacteria *E. coli* was less sensitive to tested PPEs and MBC ranged from 6.4 mg/mL to 12.8 mg/mL. In a number of studies, the pomegranate peel extracts were prepared by use of the variety of different solvents, which led to sometimes contradictory results that are very difficult to compare. Several authors reported weaker biological effect of the pomegranate water extracts in comparison with the acetone, ethanol or methanol extracts [26,27,28]. Ethanolic extracts exhibited higher degree of antibacterial activity in comparison with the ethyl acetate and butanol extracts, tested against different *E. coli* strains that cause gut infection, i.e., *E. coli* O157: H7 and the MIC value range from 0, 39 to 3, 13 mg/mL [29]. In addition, methanolic peel extracts showed a strong broad-spectrum activity against gram-positive and gram-negative bacteria, with the minimum inhibitory concentrations (MIC) ranging from 0.2 to 0.78 mg/mL [30]. Also, the 80% methanolic peels extract was a potent inhibitor of *Listeria monocytogenes*, *S. aureus*, *Escherichia coli* and *Yersinia enterocolitica* [28].

The potential use of fruit extracts and essential oils as alternatives to control growth of microorganisms and to control infectious diseases is intensively being investigated [31,32,33]. In our study, we examined the antibacterial potential of different PPEs against gram positive and gram negative bacteria and it can be concluded that ethanol extract of pomegranate peels possesses antibacterial potential against a variety of bacterial strains.

### 2.5. Statistical Analysis

**Figure 1:**
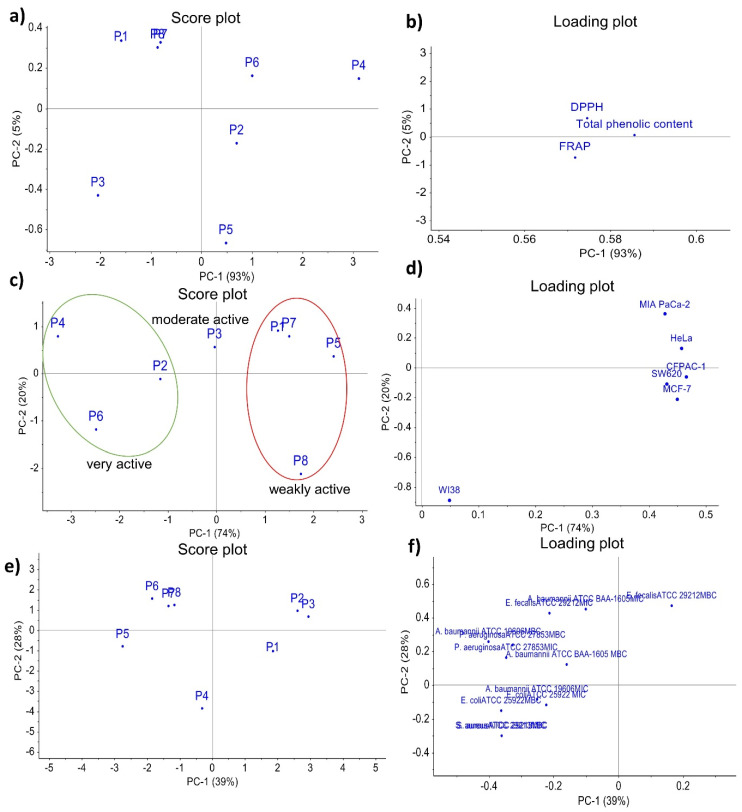
Principal component analysis (PCA) applied to different biological activities of eight pomegranate peel extracts obtained from samples P1–P8: (**a**) score plot and (**b**) loading plot when total phenolic content and antioxidant activity were used as variables; (**c**) score plot and (**d**) loading plot when antiproliferative activity was used as variables; (**e**) score plot and (**f**) loading plot when antimicrobial activity was used as variables.

A preliminary unsupervised principal component analysis (PCA) was performed to explore sample grouping based on the obtained biological activities data. In all PCA models, the observations are samples of the pomegranate peels extracts obtained from samples P1-P8, whereas different biological activity data were used as variables. In the Figure 1, the score plots on the left show sample distribution along the principal components (PCs), and loading plots on the right show the contribution of each variable to the PCs. The first two PCs were used in all models, explaining up to 98%, 94%, and 67% of the total variance in models with antioxidant activity (Figure 1a,b), antiproliferative activity (Figure 1c,d) and antimicrobial activity (Figure 1e,f) data, respectively. Observing the loading plot with antioxidant activities as variables (Figure 1a), it may be concluded that antioxidants content influenced the first PC. Therefore, samples P2, P4, P6 and P5 distributed on the right of the score plot along the positive side of PC1, can be considered as samples with higher antioxidant activity. From the loading plot with antiproliferative activity as variables (Figure 1c) it can be observed that the inhibitory concentrations (IC_50_) of pomegranate peel extracts mainly influenced the first PC. However, as lower IC_50_ means stronger antiproliferative activity, samples that are oppositely distributed from the variables can be considered as more active. Samples P2, P4 and P6 distributed along the negative side of PC1 proved to have the highest measured antiproliferative activity. Indeed, these three samples had the lowest IC_50_ values for almost all tested tumour cell lines (Table 4). The only exception was for sample P3 that showed a strong antiproliferative activity on the tumour cell line SW620.

In the both PCA models, samples P2, P4 and P6 were recognized as most active, indicating possible connection between antioxidant and antiproliferative activity on tumour cells. Additionally, it can also be observed that the antiproliferative analysis on human fibroblast data variable shows a clear segregation form other antiproliferative activities data obtained on different tumour cell lines. The last PCA model with antimicrobial activity showed that variables with MIC and MBC values mainly influenced the first PC. Once again, lower variable value indicates stronger antimicrobial activity. Therefore, considering the influence of the variables, samples P1, P2 and P3, distributed along the positive side of PC1, can be considered as the most promising samples that could have antibacterial activity against a broad range of bacteria.

Phytochemical composition of pomegranate can strongly influence biological activity of extracts. For example, stronger antioxidant capacity was connected with high-molecular-weight polyphenols ellagitannins [34]. Therefore, partial least squares (PLS) and partial least squares discriminant analysis (PLS-DA) were performed to determine metabolites that contributed the most to observed biological activity. Important variables were selected according to their variability significance at 5% levels, determined from the *p*-value for the regression coefficients. Regression coefficient describes the relationship between independent variables and the dependent variable. In two PLS analyses, response variable was one of the antioxidant activities (DPPH or FRAP), whereas predictive variables were metabolomics data. Important variables contributing positively to the antioxidant activity measured by DPPH assay were ellagic acid O-hexoside, ellagic acid and galloyl-HHDP hexose (Figure S1). As for the results of the antioxidant activity obtained by the FRAP assay, ellagic acid O-hexoside and unidentified compound (968.0160 *m*/*z*) were important variables with positive contribution (Figure S2).

Additionally, PLS-DA was used to discriminate phytochemical compounds connected with high level of antiproliferative activity. In this statistical analysis the response variable was related to the activity, i.e., dummy Y variables were assigned to samples to indicate the class of the sample as least active, moderate active and most active. The classification was performed according to previous PCA clustering. In PLS-DA with antiproliferative activity data, samples P2, P4 and P6 were classified as very active, P3 as moderate active, and all other samples as weakly active. Metabolites contributing the most to antiproliferative activity in tested samples were hexahydroxydiphenoyl (HHDP)-hexoside, ellagic acid O-hexoside and punicalin (Figure S3).

## 3. Materials and Methods

### 3.1. Samples

Wild pomegranates (*Punica granatum* L.) were collected from eight locations in western Herzegovina (Proleta branik 1, Počitelj 1, Međugorje križ, Rosića kuk 2, Proleta njiva 2 Počitelj 2, Trebižat 2, Proleta njiva 1) and were labeled as P1-P8. After harvesting, the peels were immediately separated from the grains, frozen in liquid nitrogen and stored at −20 °C for 7 days. Prior to extraction, peel samples were grounded in house blender (Mixy, Zepter International, Wollerauu, Switzerland). Plant material was identified by using usual keys and iconographies with the support of the Department of Vegetable Crops, Faculty of Agriculture, University of Zagreb.

### 3.2. Extraction of Pomegranate Peels

The extraction was carried out as follows: grounded pomegranate peels (2 g ± 0.0001) were mixed with 20 mL of 30% aqueous ethanol solution containing 1% formic acid (by volume) and extracted in an ultrasonic bath (Elmasonic S 40H, Elma, Singen, Germany) preheated to 50 °C for 30 min. Afterwards, the extracts were filtered through Whatman No. 40 filter paper (Whatman, GE Healthcare Bio-Sciences, Pittsburgh, PA, USA), transferred into volumetric flasks and made up to 25 mL volume with extraction solvent. The extraction process of each sample was done twice and extracts were stored at –20 °C in an inert nitrogen gas atmosphere until further analysis. The obtained pomegranate peel ethanol extracts (PPEs) were afterwards used for the determination of TPC, antioxidant activity and Q-TOF MS profiling. For antitumor and antibacterial assays, the ethanol extracts of pomegranate peels were evaporated to dryness using a speed vacuum (Speed Vac Plus, Savant, Waltham, MA, USA). The dry extracts were then resuspended in dimethyl sulfoxide (DMSO, Kemika, Zagreb, Croatia; 200 mg/mL) to prepare stock solutions of samples.

### 3.3. MS Analysis-Chemical Characterization

For chemical characterization, an Agilent 6550 iFunnel quadrupole time-of-flight mass spectrometer equipped with dual AJS ESI source (Agilent Technologies, Palo Alto, CA, USA) was used. MS measurements were performed as described in our previous research [35].

### 3.4. Total Phenolic Content and Antioxidant Activity Assays

#### 3.4.1. Determination of Total Phenolic Content (TPC)

Total phenolic content (TPC) was determined using a modified Folin-Ciocalteu colorimetric method described by Repajić et al. [36]. TPC were calculated according to the calibration curve for gallic acid and was expressed on a fresh weight basis as mg gallic acid equivalents (GAE)/100 g of sample.

#### 3.4.2. FRAP (Ferric Reducing Antioxidant Power) Assay

The ferric reducing antioxidant power assay was conducted using a modified FRAP method described by Dragović-Uzelac et al. [37]. The method was based on the reduction of the ferric tripyridyltriazine (Fe^3+^ TPTZ) complex to the ferrous form at low pH. Results were calculated according to the calibration curve for Trolox and expressed as µmol Trolox equivalent (TE)/100 g of fresh weight of sample.

#### 3.4.3. DPPH (2,2-Diphenyl-2-Picrylhydrazyl) Radical Scavenging Activity

The free radical scavenging capacity of pomegranate peel extracts was determined according to the procedure described by Dragović-Uzelac et al. (2010) [37] with some modifications. Results were calculated according to the calibration curve for Trolox and expressed as µmol Trolox equivalent (TE)/100 g of fresh weight of sample.

### 3.5. Antiproliferative Activity Assay

The ATCC (American Type Culture Collection, Manassas, VA, USA) human cell lines, WI-38 (normal human fibroblasts), HeLa (cervical carcinoma), SW620 (colorectal adenocarcinoma, metastatic), MCF-7 (breast epithelial adenocarcinoma, metastatic), MIA PaCa-2 (pancreatic carcinoma) and CFPAC-1 (pancreatic adenocarcinoma, derived from metastatic liver) were used for determination of antiproliferative activity of pomegranate peel extracts with the method described previously [38]. On day 0, the panel of cell lines at 3000–5000 cells per well were inoculated into a 96-well microtiter flat bottom plates FalconTM (Becton Dickinson, Franklin Lakes, NJ, USA) according to the doubling times. After 24 h cells were treated with freshly prepared samples in Dulbecco′s modified Eagle medium (DMEM; Lonza, Verviers, Belgium) in five different final concentrations ranging from 0.01 to 1.00 mg/mL (prepared from stock solution of samples in DMSO). Cell viability was determined by the use of 3-(4,5-dimethylthiazol-2-yl)-2,5-diphenyltetrazolium bromide (MTT; Sigma-Aldrich, St. Louis, MO, USA) colorimetric assay. Experimentally obtained absorbance were transformed into cell percentage growth (PG) using the formulas proposed by National Institutes of Health (NIH; Bethesda, MD, USA) [39]. The IC_50_ (concentration causing 50% growth inhibition) values for PPEs were calculated from concentration–response curves using linear regression analysis by fitting the test concentrations that give PG values above and below the reference value. Each test point was performed in quadruplicate in three individual experiments.

### 3.6. Antimicrobial Activity Assay

#### 3.6.1. Bacterial Strains and Preparation of Bacterial Suspension

The standard laboratory strains *A. baumannii* (ATCC 19606 and ATCC BAA–1605), *S. aureus* ATCC 25923, *P. aeruginosa* ATCC 27853, *E. coli* ATCC 25922, *E. fecalis* ATCC 29212, *L. monocytogenes* ATCC 19115 from culture collection of the Department of Microbiology and Parasitology, University of Rijeka were used in this study. The bacteria were stored at −80 °C in glycerol broth (10% glycerol) (Biolife, Milano, Italy).

For the assay, bacteria were cultured at 37 °C for 24 h on Mueller–Hinton broth (MHB) (Difco, Sparks, MD, USA) and 120 rpm (Unimax 1010, Heidolph, Schwabach, Germany). The optical density of the bacterial suspension was additionally estimated using a spectrophotometer at 550 nm (Eppendorf BioPhotometer, Hamburg, Germany), and number of bacterial cells was extrapolated from a standard growth curve. The viable bacterial count used in experiments was obtained by plating 10-fold dilutions onto blood agar (Biolife). After incubating plates for 24 h at 37 °C, the number of bacteria was calculated as colony forming units (CFU/mL).

#### 3.6.2. Antimicrobial Activity Testing

The broth-dilution method was conducted to determine the minimal inhibitory concentration (MIC) and the minimal bactericidal concentration (MBC) of PPEs according to the procedure described by Jurica et al. [40]. Susceptibility test was made according to Clinical and Laboratory Standards Institute (CLSI; Wayne, PA, USA) guidelines. Two-fold serial dilutions in MHB were prepared from stock solutions of each sample to give final concentrations ranging from 0.02 to 12.8 mg/mL. The inoculum of each test bacterium was prepared by diluting the overnight culture of the bacterium in MHB to a level of 1.5 × 10^7^ CFU/mL. MIC was defined as the lowest concentration of tested samples without visible growth, whereas MBC was defined as the lowest concentration of tested samples yielding negative subcultures on the solid medium.

### 3.7. Statistical Data Processing

MS data mining was performed in Mass Hunter workstation software (Agilent Technologies, Palo Alto, CA, USA)—Qualitative analysis (version B.07.00) using the molecular feature function: retention time (RT) range 0.1–2 min and *m*/*z* range 50–1200, with minimum absolute abundance of 5000 counts. Identification of compounds was obtained by comparing specific fragments of each detected compound with those in the several databases: Mass Hunter PCDL Manager (Version B.04.00), MassBank [41], mzCloud [42] or with the literature [3,10,11,12,13,14,15] Mass Profiler Professional (version 13.0) also from Mass Hunter workstation software was used for alignment, filtering and normalization of obtained MS data. Principal component analysis (PCA), Partial least square (PLS) analysis and PLS discriminant analysis (PLS-DA) was performed using UNSCRAMBLER software version 10.4 (Computer Aided Modeling (CAMO), Trondheim, Norway). Detailed procedure for statistical analysis is described in our previous research [43].

## 4. Conclusions

The present study showed that tested ethanol extracts of pomegranate peels exhibit promising antioxidant, antimicrobial and antiproliferative activities. By combining the biological tests, untargeted MS analysis and afterwards chemometrics processing, we were able to detect and identify specific compounds from PPEs that contributed the most to observed specific biological activities. Accordingly, for antioxidant activity measured by DPPH, responsible compounds were ellagic acid O-hexoside, ellagic acid and galloyl-HHDP hexose. For the antioxidant activity (obtained by the FRAP method), presence of the ellagic acid O-hexoside from identified compounds was crucial. Additionally, PLS-DA analysis revealed that metabolites contributing the most to antiproliferative activity were hexahydroxydiphenoyl (HHDP)-hexoside, ellagic acid O-hexoside and punicalin. These results are a valuable contribution to the knowledge required for exploitation of the pomegranate peels, either as a source of small molecules for a biological application or as a whole extract that could be used in different functional foods.

## Figures and Tables

**Table 1:** Compounds characteristic for ethanol pomegranate (*Punica granatum* L.) peel extracts. The table presents the experimental monoisotopic mass of the identified compound (M), molecular formula, precursor ion *m*/*z*, mass error (ppm) and fragment ions *m*/*z*.

Tentative Identification	M	Molecular Formula	*m*/*z* Precursor	Error/ppm	*m*/*z* Fragment Ions	
Citric acid	192.0270	C_6_H_8_O_7_	191.0206 (-)	−4.67	111.0089, 87.0092	
Ellagic acid	302.0063	C_14_H_6_O_8_	300.9991 (-)	−0.41	257.0040, 228.9988, 145.0266	
Epigallocatechin	306.0740	C_15_H_14_O_7_	305.0670 (-)	−1.11	139.0370, 125.0222	
Galloyl hexose	332.0743	C_13_H_16_O_10_	331.0677 (-)	−1.90	169.0090, 71.0162	
Ellagic acid O-pentoside	434.0485	C_19_H_14_O_12_	433.0413 (-)	−0.16	300.9822, 299.9749	
Ellagic acid O-hexoside	464.0591	C_20_H_16_O_13_	463.0517 (-)	0.29	300.9845, 299.9763	
Hexahydroxydiphenoyl (HHDP)-hexoside	482.0697	C_20_H_18_O_14_	481.0622 (-)	0.32	300.9802, 275.0057	
Digalloyl hexose	484.0853	C_20_H_20_O_14_	483.0777 (-)	0.68	331.0461, 271.0339, 169.0097	
Galloyl-HHDP hexose	634.0806	C_27_H_22_O_18_	633.0731 (-)	0.37	362.9182, 300.9809, 275.0061	
Lergastannin C	650.0755	C_27_H_22_O_19_	649.0965 (-)	−1.92	300.9841, 191.0130	
Punicalin	782.0603	C_34_H_22_O_22_	781.0539 (-)	−1.16	600.9501, 300.9854, 275.0084	
Pedunculagin I	784.0759	C_34_H_24_O_22_	783.0698 (-)	−1.42	300.9810, 275.0050	
Peducalangin II	785.0832	C_34_H_24_O_22_	785.0848 (-)	−2.04	633.0165, 300.9799, 275.0057	
Peducalangin III	934.0712	C_41_H_26_O_26_	933.0643 (-)	−0.37	783.0121, 720.9766, 600.9431, 300.9826, 275.0057, 191.0153	
Casuarinin	936.0869	C_41_H_28_O_26_	935.0782 (-)	1.30	633.0200, 331.0475, 300.9801, 275.0032	
Granatin like	938.1025	C_41_H_30_O_26_	937.0939 (-)	1.41	787.0197, 785.0197, 300.9799	
Granatin B	952.0818	C_41_H_28_O_27_	951.0753 (-)	−0.87	783.0008, 605.02841, 300.98300	
Punicalagin derivative	980.1138	C_43_H_32_O_27_	979.1065 (-)	−0.67	300.9834, 61.9912	
Punicalagin α Punicalagin β	1084.0665	C_45_H_28_O_30_	1083.0603 (-)	−0.98	780.9933, 600.9470, 450.9654	

**Table 2:** Presence of tentatively identified bioactive compounds in eight ethanol pomegranate *(Punica granatum* L.) peel extracts (PPEs).

Compound	Sample							
	P1	P2	P3	P4	P5	P6	P7	P8
Casuarinin	+	-	+	+	+	-	-	-
Citric acid	+	+	+	+	+	+	+	+
Digalloyl hexose	-	+	+	-	-	+	+	-
Ellagic acid	+	+	+	+	+	+	+	+
Ellagic acid O-hexoside	+	+	+	+	+	+	+	+
Ellagic acid O-pentoside	+	+	+	+	+	+	+	+
Epigallocatechin	-	+	+	+	-	-	+	-
Galloyl hexose	+	+	+	+	+	+	+	+
Galloyl-HHDP hexose	+	+	+	+	+	+	+	+
Granatin B	+	+	+	+	+	+	+	+
Granatin like	-	+	-	-	-	+	+	-
Hexahydroxydiphenoyl (HHDP)-hexoside	+	+	+	+	+	+	+	+
Lergastannin C	-	+	-	-	-	+	-	-
Peducalangin II	-	+	-	-	-	+	+	-
Peducalangin III	+	+	-	+	-	+	-	-
Pedunculagin I	+	+	+	+	+	+	+	+
Punicalagin derivative	+	+	+	+	+	+	+	+
Punicalagin α Punicalagin β	+ +	+ +	+ +	+ +	+ +	+ +	+ +	+ +
Punicalin	+	+	+	+	+	+	+	+

**Table 3:** The content of total polyphenols and antioxidant activity (DPPH and FRAP values) of ethanol extracts of pomegranate *(Punica granatum* L.) peels (PPEs). Result are given as a mean value ± standard deviation (SD).

Sample	Total Polyphenols (mg/100 g) ^1^	DPPH (µmol/100 g) ^2^	FRAP (μmol/100 g) ^2^
P1	6390.56 ± 185.52	7593.40 ± 39.16	100.25 ± 0.26
P2	7761.07 ± 167.50	7705.85 ± 12.62	150.53 ± 14.24
P3	5766.44 ± 114.86	7525.42 ± 70.71	109.36 ± 11.38
P4	10599.43 ± 17.57	7831.42 ± 12.59	176.60 ± 3.47
P5	7575.95 ± 26.50	7661.62 ± 41.66	156.62 ± 8.61
P6	8028.04 ± 8.84	7744.40 ± 64.43	148.82 ± 7.19
P7	6360.16 ± 26.48	7659.99 ± 26.58	117.94 ± 3.92
P8	6402.71 ± 284.89	7651.31 ± 18.59	116.69 ± 16.08

^1^ Results are expressed as the gallic acid equivalents ((GAE)/100 g of sample). ^2^ Results are expressed as the Trolox equivalent ((TE)/100 g of fresh weight of sample).

**Table 4:** The inhibitory concentrations (IC_50_) of ethanol extracts (PPEs) of pomegranate *(Punica granatum* L.) peels on normal human fibroblasts (WI38), cervical carcinoma (HeLa), colorectal adenocarcinoma, metastatic (SW620), breast epithelial adenocarcinoma, metastatic (MCF-7), pancreatic carcinoma (MIA PaCa-2) and pancreatic adenocarcinoma, derived from metastatic liver (CFPAC-1).

Sample	IC_50_^1^					
	WI-38	HeLa	SW620	MCF-7	MIA PaCa-2	CFPAC-1
P1	0.197	0.212	0.255	0.239	0.219	0.334
P2	0.245	0.167	0.223	0.198	0.164	0.274
P3	0.205	0.185	0.208	0.219	0.191	0.331
P4	0.151	0.141	0.163	0.162	0.136	0.197
P5	0.214	0.212	0.354	0.272	0.216	0.365
P6	0.319	0.145	0.209	0.173	0.132	0.234
P7	0.160	0.191	0.317	0.268	0.203	0.353
P8	0.396	0.196	0.304	0.303	0.177	0.370

**^1^** IC_50_—Concentration (mg/mL) required for inhibition of cancer cell proliferation by 50%.

**Table 5:** The minimum inhibitory concentrations (MICs) and the minimum bactericidal concentrations (MBCs) of ethanol extracts of pomegranate *(Punica granatum* L.) peels on different bacteria.

Sample	*A. baumannii* ATCC 19606		*A. baumannii* ATCC BAA-1605		*S. aureus* ATCC 25923		*P. aeruginosa* ATCC 27853		*E. coli* ATCC 25922		*E. fecalis* ATCC 29212		*L. monocytogenes* ATCC 19115	
	MIC ^1^	MBC ^2^	MIC ^1^	MBC ^2^	MIC ^1^	MBC ^2^	MIC ^1^	MBC ^2^	MIC ^1^	MBC ^2^	MIC ^1^	MBC ^2^	MIC ^1^	MIC ^2^
P1	6.4	6.4	6.4	6.4	0.8	0.8	6.4	6.4	12.8	12.8	3.2	6.4	12.8	12.8
P2	6.4	6.4	12.8	>12.8	0.8	0.8	6.4	6.4	6.4	6.4	3.2	6.4	12.8	12.8
P3	3.2	6.4	12.8	>12.8	0.8	0.8	6.4	3.2	12.8	6.4	3.2	6.4	12.8	12.8
P4	6.4	6.4	6.4	12.8	6.4	6.4	6.4	3.2	12.8	12.8	1.6	1.6	12.8	12.8
P5	6.4	12.8	12.8	>12.8	6.4	6.4	12.8	12.8	12.8	12.8	3.2	3.2	12.8	12.8
P6	6.4	12.8	12.8	12.8	3.2	3.2	12.8	12.8	12.8	12.8	6.4	6.4	12.8	12.8
P7	6.4	12.8	12.8	>12.8	3.2	3.2	12.8	6.4	12.8	12.8	6.4	6.4	12.8	12.8
P8	6.4	12.8	12.8	>12.8	3.2	3.2	6.4	12.8	12.8	12.8	6.4	6.4	12.8	12.8

**^1^** MIC—Concentration (mg/mL) required for 99% bacteriostatic effect. **^2^** MBC—Concentration (mg/mL) required for 99% of bacterial killing effect.

## Supplementary Materials

Figure S1. Weighted regression coefficients plot with marked significant predictive variables obtained from the PLS analysis with DPPH antioxidant activity as response variable. If variable disrespect has uncertainty limits crossing the zero line, it is not significant at the 5% level. Ellagic acid O-hexoside, ellagic acid and galloyl-HHDP hexose are found to be significant at the 5% level and positively contribute to the antioxidant activity. Figure S2. Weighted regression coefficients plot with marked significant predictive variables obtained from the PLS analysis with FRAP antioxidant activity as response variable. If variable disrespect has uncertainty limits crossing the zero line, it is not significant at the 5% level. Ellagic acid O-hexoside and unidentified compound with mass 968.0160 are found to be significant at the 5% level and positively contribute to the antioxidant activity. Figure S3. Weighted regression coefficients plot with marked significant predictive variables obtained from the PLS-DA analysis with antiproliferative activity as response variable. If variable disrespect has uncertainty limits crossing the zero line, it is not significant at the 5% level. Hexahydroxydiphenoyl (HHDP)-hexoside, ellagic acid O-hexoside and punicalin are found to be significant at the 5% level and positively contribute to the overall antiproliferative activity.
