# Supplementary material for: Assessment of the Biological Activity and Phenolic Composition of Ethanol Extracts of Pomegranate (Punica granatum L.) Peels
Source: Molecules. 2020 Dec 14;25(24):5916. doi: 10.3390/molecules25245916 (PMC7764994; doi:10.3390/molecules25245916)
Supplement: Supplementary file 1 [file molecules-25-05916-s001.pdf]

Supplementary Material

Assessment of the Biological Activity and Phenolic Composition of Ethanol Extracts of Pomegranate (*Punica granatum* L.) Peels

Željka Peršurić [1], Lara Saftić Martinović [1], Mladenka Malenica [2], Ivana Gobin [2], Sandra Pedisić [3], Verica Dragović-Uzelac [3] and Sandra Kraljević Pavelić [4,*]

[1] Department of Biotechnology, University of Rijeka, Radmile Matejčić 2, HR-51000 Rijeka, Croatia; zpersuric@biotech.uniri.hr (Ž.P.), lara.saftic@biotech.uniri.hr (L.S.M.),
[2] Faculty of Medicine, University of Rijeka, Braće Branchetta 20, HR-51000 Rijeka, Croatia; mladenkams@medri.uniri.hr (M.M.), ivana.gobin@medri.uniri.hr (I.G.)
[3] Faculty of Food Technology and Biotechnology, University of Zagreb, Pierottijeva 6, HR-10000 Zagreb, Croatia; spedisic@pbf.hr (S.P.), vdragov@pbf.hr (V.D.-U.)
[4] Faculty of Health Studies, University of Rijeka, Viktora Cara Emina 5, HR-51000 Rijeka, Croatia; sandrakp@uniri.hr (S.K.P.)
* Correspondence: sandrakp@uniri.hr

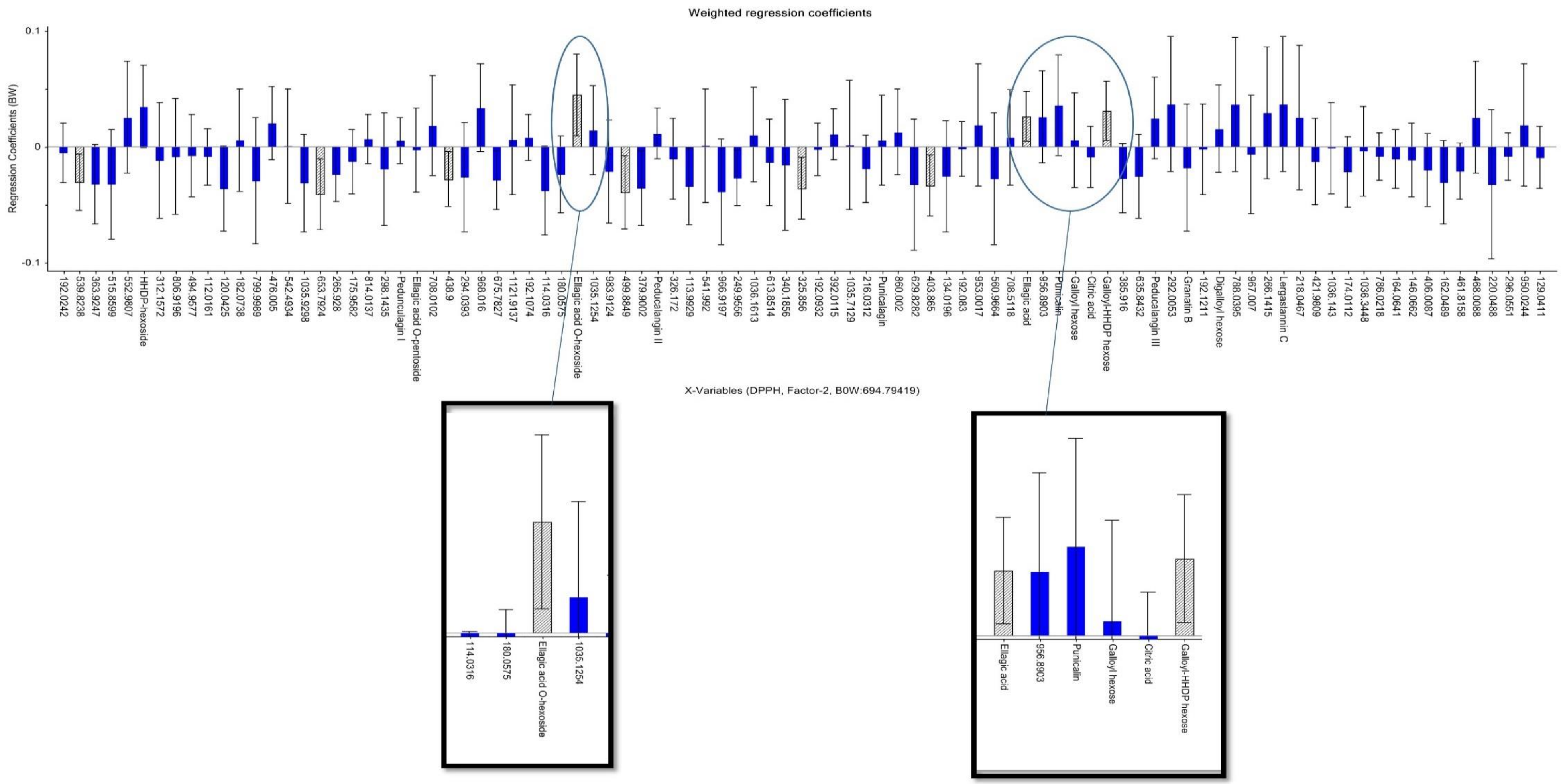

Figure S1. Weighted regression coefficients plot with marked significant predictive variables obtained from the PLS analysis with DPPH antioxidant activity as response variable. If variable disrespect has uncertainty limits crossing the zero line, it is not significant at the 5% level. Ellagic acid O-hexoside, ellagic acid and galloyl-HHDP hexose are found to be significant at the 5% level and positively contribute to the antioxidant activity.

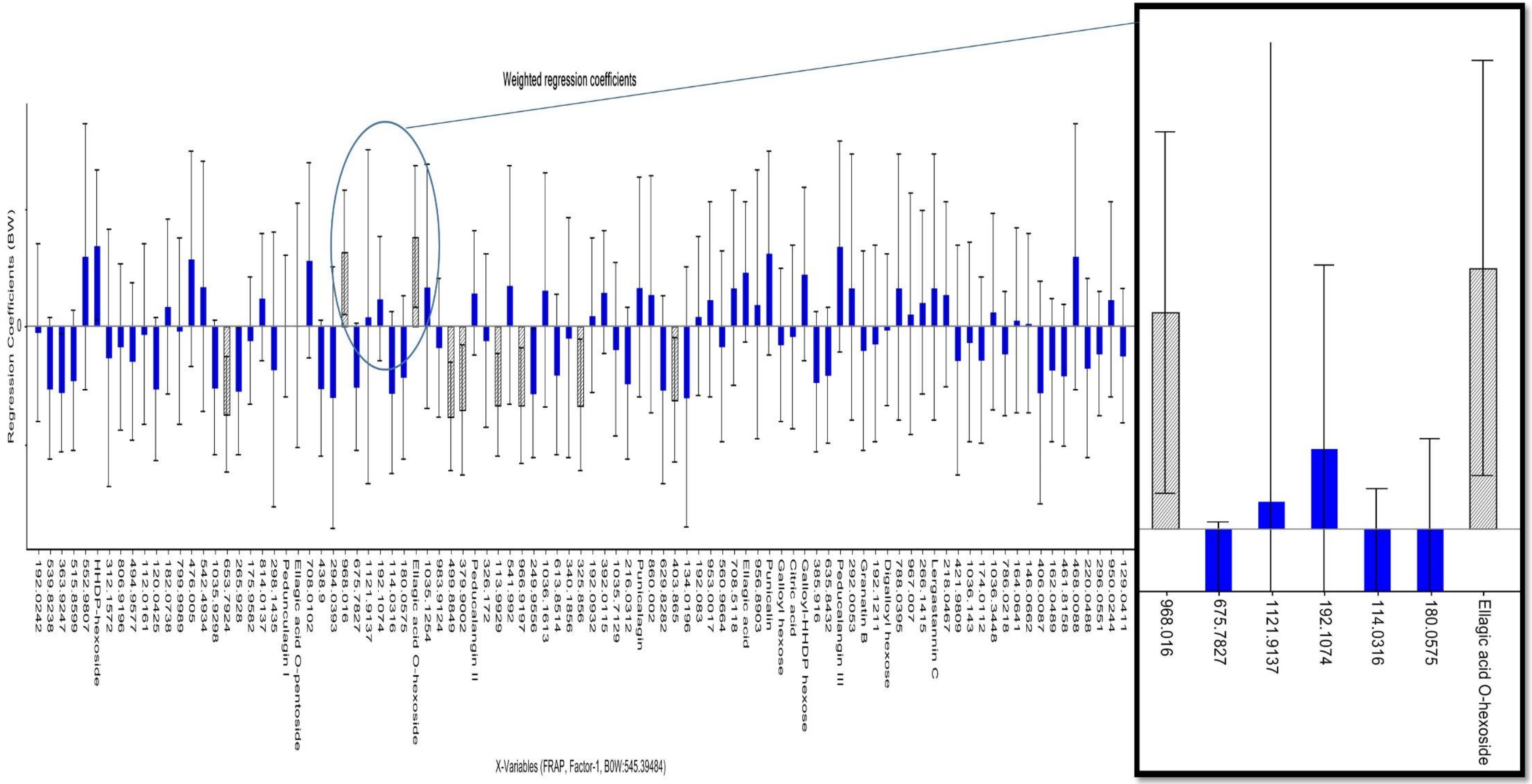

Figure S2. Weighted regression coefficients plot with marked significant predictive variables obtained from the PLS analysis with FRAP antioxidant activity as response variable. If variable disrespect has uncertainty limits crossing the zero line, it is not significant at the 5% level. Ellagic acid O-hexoside and unidentified compound with mass 968.0160 are found to be significant at the 5% level and positively contribute to the antioxidant activity.

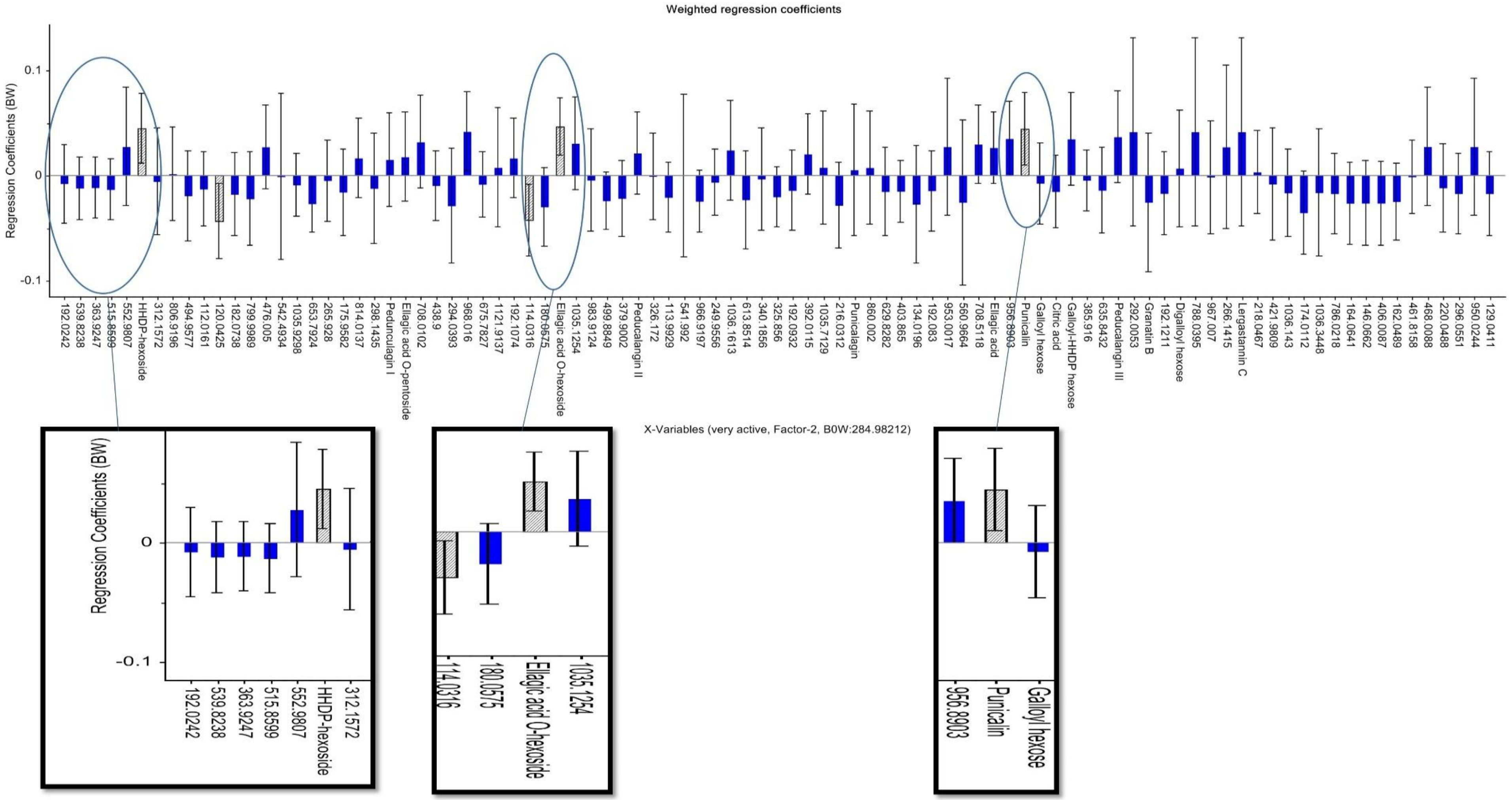

Figure S3. Weighted regression coefficients plot with marked significant predictive variables obtained from the PLS-DA analysis with antiproliferative activity as response variable. If variable disrespect has uncertainty limits crossing the zero line, it is not significant at the 5% level. Hexahydroxydiphenoyl (HHDP)-hexoside, ellagic acid O-hexoside and punicalin are found to be significant at the 5% level and positively contribute to the overall antiproliferative activity.
